# Mobilizing Patient and Public Involvement in the Development of Real-World Digital Technology Solutions: Tutorial

**DOI:** 10.2196/44206

**Published:** 2023-10-27

**Authors:** Alison Keogh, Ríona Mc Ardle, Mara Gabriela Diaconu, Nadir Ammour, Valdo Arnera, Federica Balzani, Gavin Brittain, Ellen Buckley, Sara Buttery, Alma Cantu, Solange Corriol-Rohou, Laura Delgado-Ortiz, Jacques Duysens, Tom Forman-Hardy, Tova Gur-Arieh, Dominique Hamerlijnck, John Linnell, Letizia Leocani, Tom McQuillan, Isabel Neatrour, Ashley Polhemus, Werner Remmele, Isabel Saraiva, Kirsty Scott, Norman Sutton, Koen van den Brande, Beatrix Vereijken, Martin Wohlrab, Lynn Rochester, Mobilise-D consortium

**Affiliations:** 1 Insight Centre Data Analytics University College Dublin Dublin4 Ireland; 2 School of Medicine Trinity College Dublin Dublin2 Ireland; 3 Translational and Clinical Research Institute, Faculty of Medical Sciences Newcastle University Newcastle United Kingdom; 4 Department of Neuromedicine and Movement Science Faculty of Medicine and Health Sciences Norwegian University of Science and Technology Trondheim Norway; 5 Clinical Science and Operations Global Development Sanofi Research & Development Chilly-Mazarin France; 6 Clario Clario Holdings Inc Geneva Switzerland; 7 Mobilise-D Patient and Public Advisory Group Newcastle United Kingdom; 8 Department of Clinical Neurology Sheffield Teaching Hospitals National Health Service Foundation Trust Sheffield United Kingdom; 9 Sheffield Institute for Translational Neuroscience The University of Sheffield Sheffield United Kingdom; 10 Department of Mechanical Engineering University of Sheffield Sheffield United Kingdom; 11 Insigneo Institute for in Silico Medicine University of Sheffield Sheffield United Kingdom; 12 National Heart and Lung Institute Imperial College London London United Kingdom; 13 School of Computer Science Newcastle University Newcastle United Kingdom; 14 AstraZeneca Research and Development Global Regulatory Excellence Paris France; 15 Non-Communicable Diseases and Environment Programme ISGlobal Barcelona Spain; 16 Department of Medicine and Life Sciences Universitat Pompeu Fabra Barcelona Spain; 17 Centro de Investigación Biomedical en Red Epidemiologia y Salud Publica Barcelona Spain; 18 Department of Neurology San Raffele University Milan Italy; 19 Epidemiology, Biostatistics and Prevention Institute University of Zurich Zurich Switzerland; 20 Dr. Margarete Fischer-Bosch-Institute of Clinical Pharmacology Stuttgart Germany; 21 University of Tuebingen Tuebingen Germany; 22 Newcastle Upon Tyne Hospitals National Health Service Foundation Trust Newcastle United Kingdom; 23 See Acknowledgments

**Keywords:** patient involvement, patient engagement, public-private partnership, research consortium, digital mobility outcomes, real-world mobility, digital mobility measures

## Abstract

Although the value of patient and public involvement and engagement (PPIE) activities in the development of new interventions and tools is well known, little guidance exists on how to perform these activities in a meaningful way. This is particularly true within large research consortia that target multiple objectives, include multiple patient groups, and work across many countries. Without clear guidance, there is a risk that PPIE may not capture patient opinions and needs correctly, thereby reducing the usefulness and effectiveness of new tools. Mobilise-D is an example of a large research consortium that aims to develop new digital outcome measures for real-world walking in 4 patient cohorts. Mobility is an important indicator of physical health. As such, there is potential clinical value in being able to accurately measure a person’s mobility in their daily life environment to help researchers and clinicians better track changes and patterns in a person’s daily life and activities. To achieve this, there is a need to create new ways of measuring walking. Recent advancements in digital technology help researchers meet this need. However, before any new measure can be used, researchers, health care professionals, and regulators need to know that the digital method is accurate and both accepted by and produces meaningful outcomes for patients and clinicians. Therefore, this paper outlines how PPIE structures were developed in the Mobilise-D consortium, providing details about the steps taken to implement PPIE, the experiences PPIE contributors had within this process, the lessons learned from the experiences, and recommendations for others who may want to do similar work in the future. The work outlined in this paper provided the Mobilise-D consortium with a foundation from which future PPIE tasks can be created and managed with clearly defined collaboration between researchers and patient representatives across Europe. This paper provides guidance on the work required to set up PPIE structures within a large consortium to promote and support the creation of meaningful and efficient PPIE related to the development of digital mobility outcomes.

## Introduction

### Background

Patient and public involvement and engagement (PPIE), defined as “research being carried out ‘with’ or ‘by’ members of the public (including patients and carers) rather than ‘to’, ‘about’ or ‘for’ them” [1,2], has been emphasized as an integral part of the research process by multiple health agencies and funding bodies [3-6]. This is because of its importance in creating meaningful research studies and outcomes across diverse patient needs. PPIE may occur in multiple contexts and stages ranging from strategic to operational to local to national [7], and PPIE activities may cover a range of topics, including acceptability, data sharing, visualizations of complex data, dissemination methods, and protocols. PPIE is argued to be a basic right within the research process [8], which helps make it more relevant to the public [5,8,9]. Inclusive and diverse PPIE activities are important in the development of tools and interventions to better address users’ needs and increase usability [10-12].

Despite recommendations, PPIE reporting is inconsistent [13], and the methodology remains unclear or lacking [9,14]. Published examples typically focus on discrete tasks or research with single patient groups only [15-18]. Furthermore, existing guidelines fail to provide sufficient detail on how PPIE should be conducted in a meaningful manner [19,20], mostly because of the specificity and unique needs of each project. Nonetheless, the guidance provided by existing recommendations [20,21] is not sufficiently detailed to address the complexity inherent in multicohort consortia with multiple research objectives [20,21]. Engagement in such large projects requires PPIE structures and contributors that consider and respond to the cultural and cohort-specific differences of advisers in a way that aligns them all toward the project objectives. Furthermore, such projects tend to be long in duration (>4 years), meaning that the structures need to be robust and long enough to respond to required changes in protocols or circumstances while still maintaining solid PPIE engagement that aligns with the scope of the project. Without clear guidance, PPIE activities in these consortia risk being just *box ticking* exercises that fail to adequately address patient needs [22,23] and may contribute to exclusion in trials. For example, digital tools developed without patient insight may be too difficult to use or inaccessible, creating inequities, whereas recruitment strategies designed without input from a diverse group of public contributors may fail to consider the needs of underserved participants and, therefore, impose barriers to participation in research trials [24]. Granted, guidance cannot and should not be prescriptive in nature [9,19,20]; however, there is a clear need for further research and transparency around experiences in this area so that project stakeholders can share knowledge, improve methodology, and ensure that PPIE is embedded in a meaningful manner [9,13,14].

**Figure 1:**
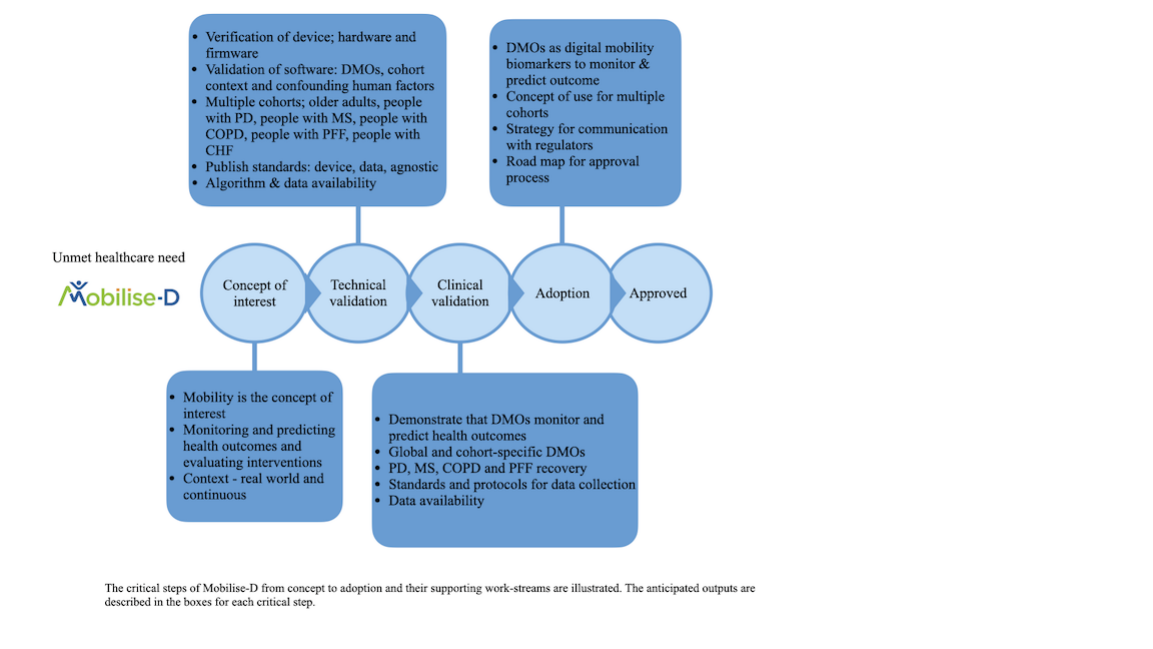
Mobilise-D road map (adapted from Rochester et al). CHF: chronic heart failure; COPD: chronic obstructive pulmonary disorder; DMO: digital mobility outcome; MS: multiple sclerosis; PD: Parkinson disease; PFF: proximal femoral fracture.

One such area in which PPIE work has the potential to be hugely impactful is the measurement of mobility. Mobility is heralded as the sixth vital sign [25], as it is a significant indicator of mortality and quality of life [26-29]. Measuring mobility in the real world is a complex task that requires significant clinical and technical expertise [30], which would greatly benefit from the insights of patients with chronic conditions that impact their mobility. Such engagement would support the development of improved measurement tools that not only are technically accurate but also reflect the aspects of mobility that are important to people. Nonetheless, because of the technical complexity of the quantification of a person’s mobility, it continues to rely on laboratory-based assessments or self-reported outcomes [30,31]. However, the ongoing digitization within health care could revolutionize how real-world mobility is tracked and evaluated. Specifically, this paper focuses on an example of such digital innovation. Mobilise-D is a public-private partnership funded by the European Innovative Medicines Initiative 2 Joint Undertaking [31]. Its overarching objective is to develop and validate new digital mobility outcomes with the aim of gaining regulatory approval in a variety of disease states, including Parkinson disease, chronic obstructive pulmonary disease, multiple sclerosis, and recovery from proximal femoral fracture. The associated research program (Figure 1 [31]) spans from 2019 to 2024 and incorporates a technical validation study (TVS) and clinical validation study (CVS) of the targeted digital mobility outcome measures. The TVS was conducted between 2019 and 2021. It adopted a multifaceted and multidisciplinary approach aimed at (1) verifying the metrological performance of the included sensors, (2) establishing the technical validity of the algorithms used to estimate digital mobility outcomes using wearable sensor data, and (3) establishing the acceptability of the deployed sensor. The CVS is being conducted between 2021 and 2024 and will demonstrate that the selected digital mobility outcomes quantified using the algorithms validated by Mobilise-D measure what they aim to measure, are clinically meaningful to patients and clinicians, and can evaluate changes over time.

When creating new digital solutions, it is important to ensure that they are clinically meaningful, are impactful, are inclusive of diverse groups, and respond to the needs of these groups accordingly [6,10,32,33]. This evidence is also required for regulatory approval, which is a key aim of the Mobilise-D consortium [34-36]. Therefore, PPIE activities are fundamental to the development of digital mobility solutions in general and within Mobilise-D specifically. The insights derived from representatives from multiple patient cohorts on this complex topic are integral to the ultimate success of these solutions if they are to be approved for use and used widely in the future.

### Aims

Consequently, in this tutorial, we aimed to describe, in detail, the PPIE approach adopted by the Mobilise-D consortium. Through this tutorial, we provide guidance on how to implement PPIE activities in a large multidisciplinary consortium. Specifically, we do this through the following steps, which outline the iterative and evolutionary nature of our work:

- Describe the evolution and implementation of a comprehensive PPIE strategic framework and associated activities to support the multistakeholder, multiobjective activities of Mobilise-D
- Describe the contributions and impact of the work of patient advisers within the consortium
- Assess the impact of being part of a consortium from the perspective of patient advisers
- Identify lessons learned and key recommendations for PPIE implementation based on the experiences and details derived from the aforementioned objectives

### The Evolution and Implementation of a PPIE Framework Within Mobilise-D

#### Recognizing the Need for a Consortium-Wide Strategic Approach

**Figure 2:**
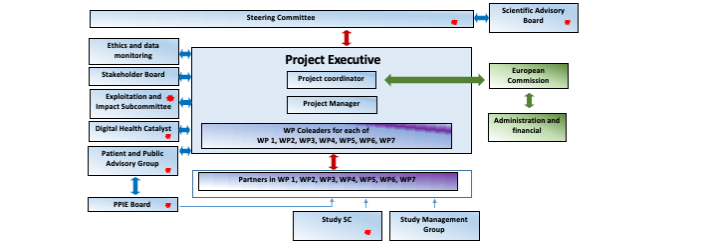
Project management structure of Mobilise-D. PPIE: patient and public involvement and engagement; SC: Steering Committee; WP: work package (Exploitation and Impact Subcommittee, Scientific Advisory Board, Study SC, Digital Health Catalyst, PPIE Board, and Patient and Public Advisory Group had patient or public representation).

In developing a body of work that centers on a person’s mobility in their daily environment and mobility across multiple patient cohorts, Mobilise-D aims to tackle multiple challenges at once, requiring approaches across the domains of governance, research, and dissemination. Specifically, the research objectives of the consortium are to (1) define optimal digital mobility outcomes, (2) support and determine the clinical validation of digital mobility outcomes, (3) build a platform for robust digital data management, (4) define and set standards for technology-unbiased digital mobility assessment, (5) create an enduring impact by establishing the largest biobank of digital mobility data, and (6) ensure the dissemination and sustainability of the project. This work was split among 7 different work packages, each of which was responsible for a particular area, such as TVS, CVS, data management, data analysis, project management, and dissemination (Figure 2). The strength of the consortium is that it brings together people with a range of chronic conditions to consider a single yet crucial aspect of health, the ability to move around. Although this allows for a variety of insights from people with different lived experiences and sociodemographic characteristics, it also means that significant effort is required to ensure collaborative work across disciplines and stakeholders.

**Figure 3:**
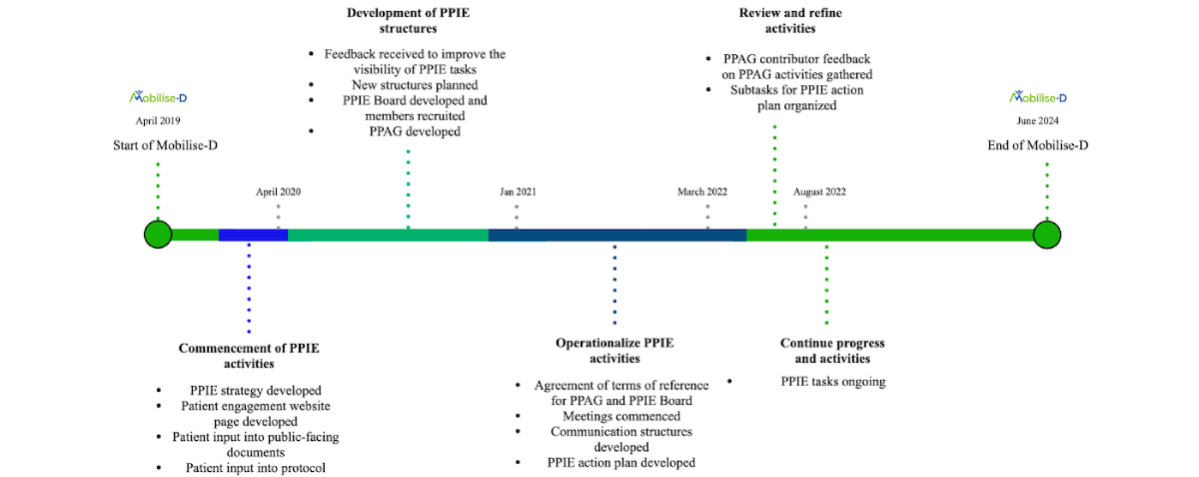
Timeline of patient and public involvement and engagement (PPIE) activities within Mobilise-D. PPAG: Patient and Public Advisory Group.

From the outset, PPIE activities were ingrained within Mobilise-D (Figure 3) [31]. Early tasks included developing a PPIE framework, seeking patient input on the study protocol, gathering feedback on patient-facing information documents, providing newsletters to study participants, presenting Mobilise-D to patient groups, and developing a dedicated patient engagement page on the Mobilise-D website. However, it became clear that these activities should be better connected to core research objectives and should increase their visibility to the public and wider consortium members. Without this, the strategic framework was not able to move forward as intended. Indeed, within the first year of the consortium, the complexity of PPIE tasks turned out to be much greater than anticipated. Thus, it became clear that meaningful PPIE work would require a more integrated approach with a dedicated team to move it forward. Consequently, the strategic framework was actioned through the development of new consortium-wide structures, and a refined action plan that targeted each of the consortium objectives within the context of the complex consortium ecosystem was developed (Figure 3).

#### PPIE Principles in Mobilise-D

In our work with various stakeholders (eg, patients, clinicians, and patient organizations) and with the aim of covering multiple patient populations, cultures, and languages, we defined the following terms to distinguish between the different types of activities we perform: participation, engagement, and involvement. These are our core PPIE principles:

- Patients will be asked to *participate* directly in Mobilise-D as participants in our studies.
- We will *engage* with the public through regular updates on the research findings and the work of the consortium.
- Importantly, we will *involve* patients and representatives from the public throughout the different phases of Mobilise-D through a variety of activities, including consultation, collaboration, and the co-design of patient information leaflets and other study materials.

#### Creating PPIE Structures

##### Overview

**Figure 4:**
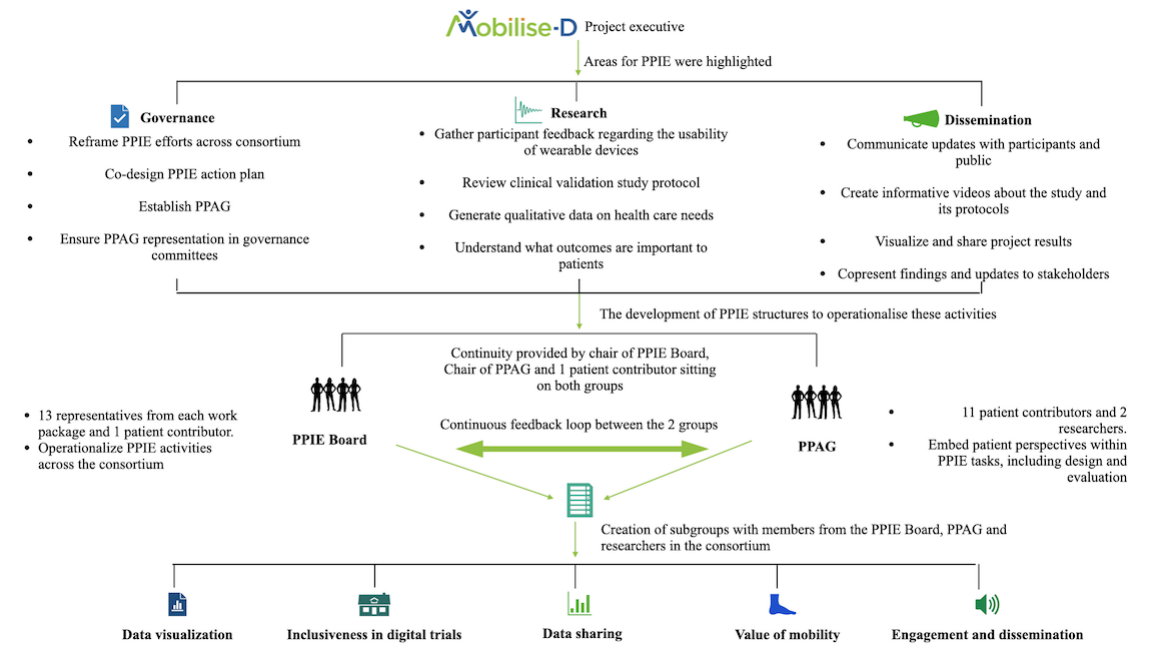
Patient and public involvement and engagement (PPIE) structures and development processes in Mobilise-D. PPAG: Patient and Public Advisory Group.

Focusing on a single aspect of health from a multidisciplinary perspective of multiple advisers requires the development of appropriate structures that can consider the individual needs of each cohort within the objectives of the consortium. Hence, the project executive created 2 new subgroups focused on overseeing and implementing the PPIE activities of the consortium, namely the PPIE Board and the Patient and Public Advisory Group (PPAG), along a specific role of PPIE lead (AK) within the consortium to facilitate interaction between the 2 groups (Figure 4). The PPIE lead is an experienced qualitative researcher with a clinical background (as a physiotherapist) pertinent to the modality of interest (ie, mobility). Their work focuses on evaluating the acceptability of digital health technology among patients and health care professionals; therefore, they are experienced in liaising with patients and stakeholders in a range of settings. These 2 groups are within the overall governance structure of the Mobilise-D consortium.

##### PPIE Board

The PPIE Board oversees and guides the implementation of PPIE activities across the consortium. Chaired by the Mobilise-D principal investigator (LR), with a European Federation of Pharmaceutical Industries and Associations partner cochair (SC-R), it consists of 13 representatives from each of the consortium work packages and a representative from the PPAG (Figure 4). The PPIE Board operationalizes the PPIE activities and ensures that they occur in line with, and to the benefit of, the consortium objectives. Initially, PPIE Board meetings were held every 2 weeks between January 2021 and June 2021 to allow structures to develop and to further expand and implement the action plan. Following this, monthly meetings were held up to January 2022, after which the meetings occurred quarterly to allow tasks to progress sufficiently between meetings.

##### PPAG Structure

The PPAG consists of 11 patient representatives from the clinical cohorts included in the Mobilise-D project along with the Mobilise-D principal investigator (LR) and the PPIE lead (AK). No carers were recruited to the group. The contributors were aged between 40 and 70 years and recruited from 8 countries, namely the United Kingdom (3/11, 27%), Belgium (2/11, 18%), Germany (1/11, 9%), Italy (1/11, 9%), Israel (1/11, 9%), the Netherlands (1/11, 9%), Portugal (1/11, 9%), and the United States (1/11, 9%). Contributors were diagnosed with Parkinson disease (4/11, 36%), chronic obstructive pulmonary disease or other chronic respiratory diseases (3/11, 27%), or multiple sclerosis (4/11, 36%). Among the 11 contributors, 4 (36%) were women, and 7 (64%) were men. All members were White and could converse well in English.

The PPAG was created to ensure that the needs and opinions of patient and public contributors are embedded throughout the project work, action plan, and its activities by identifying topics of importance, highlighting any changes required in protocols, and supporting the interpretation and dissemination of findings. The PPAG contributors agreed that they would like to meet between 2 and 4 times a year. To date, meetings typically have between 50% and 90% attendance, and if a PPAG contributor cannot attend, they are offered an individual meeting with the PPIE lead (AK) instead. In addition to sitting on the PPAG, the contributors from this group also sit on other committees throughout the governance structure of the consortium and provide their guidance to other subtasks within the PPIE action plan (Figure 3), thus ensuring regular interaction between the researchers and PPAG contributors.

##### Interaction Between the PPIE Board and the PPAG

Although the PPIE Board and PPAG meet independently, there is a crossover in membership, which supports the continuity of planning (Figure 4). The PPIE lead role was created to lead the PPIE activities of the consortium. The PPAG highlighted that they wished for this PPIE lead (AK) to act as the contact point between the PPAG and PPIE Board. Sitting on both groups, the PPIE lead ensures that there is a consistent contact point between the PPAG members and Mobilise-D consortium. This is further supported by other researchers in the consortium who either sit on or interact with both groups. Whereas the PPAG advises on and supports the development of PPIE tasks, the PPIE Board operationalizes them and ensures that they align with the consortium objectives. Thus, these 2 groups work collaboratively in a continuous feedback loop process (Figure 3).

### Developing the PPAG and Its Operations

#### Terms of Reference

It was important that both the PPIE Board and the PPAG were aware of their roles and responsibilities to support collaboration and recruit contributors who understood the expectations of each group. Thus, terms of reference and a code of conduct were drafted before the first meeting of each group and sent in advance to allow all the members to consider whether any changes were needed. This was especially relevant for the PPAG members, who were entering into the project for the first time. The terms of reference documents included (1) the aims of the group, (2) criteria for membership, (3) practical details regarding meetings, (4) planned methods of communication, (5) sections on confidentiality and responsibility, and (6) an emphasis on the important value of all opinions. In addition, a reimbursement procedure was developed and subsequently reviewed and agreed upon by the PPAG contributors and other governing committees of the consortium.

#### Membership and Recruitment

To join the PPAG, contributors needed to fulfill the criteria identified by the PPIE Board. First, potential members had to be diagnosed with one of the conditions being evaluated within Mobilise-D or be a family member or carer of someone diagnosed with one of the mentioned conditions. Next, owing to the volume of remote work that would be required, contributors had to be comfortable with the use of technology for communication (ie, email, video calls, etc). They also needed to be able to communicate in English. Although it was acknowledged that these criteria would exclude certain demographics of patients and public representatives, it was a limitation that was difficult to avoid.

Potential contributors (n=11) were identified using convenience sampling through clinicians within the consortium and through patient societies. Those who expressed an interest (n=9) in the group were contacted by the PPIE lead and invited to an initial meeting where Mobilise-D was introduced, terms of reference were agreed upon, and any questions that the members had were answered. Potential contributors were given approximately 6 weeks’ notice of the meeting and were provided with a copy of the draft terms of reference, a nondisclosure agreement, a consent form, a proposed code of conduct, and the presentation slides that would be used in the meeting and included the questions that would be asked on the day. This allowed sufficient time for the group members to reflect on any changes they felt should be made to the terms of reference and to consider any questions that they may have on the project or the work of the PPAG itself. Since its establishment, further members have been recruited, as 2 members left the group for health reasons, and a further 2 members experienced significant worsening of their condition during the first year of the group, resulting in a reduced capacity to contribute. Therefore, we approached 4 additional members between 6 months and 1 year after the PPAG was established to ensure that we maintained enough members for when further temporary or permanent absences occurred.

#### Communication

During the first PPAG meeting, which took place over videoconferencing software, various practical elements of work were discussed. Specifically, based on the preferences of the PPAG contributors, it was agreed that the PPIE implementation lead should chair the group and facilitate communication between the contributors and Mobilise-D consortium. It was also acknowledged that there would be times when the members may not have the capacity to be involved in tasks; thus, flexibility was needed to accommodate availability and input. The contributors agreed that email was their preferred contact method and that documents for discussion should be sent 1 month in advance so that the members would have sufficient notice of upcoming meetings and their content. However, the contributors noted that they also wanted to be able to discuss items away from meetings. Thus, the Voice Global platform [37] was introduced as a communication tool with a closed forum dedicated to the PPAG, where documents could be uploaded and discussed at a time suitable for each individual contributor. The purpose of Voice Global [37] was to support the PPAG members in adding their comments and suggestions at a time suitable for themselves. However, as time progressed, the PPAG contributors demonstrated different preferences in how they provided feedback and were communicated with. Thus, we adjusted this step further in line with their preferences. The Voice Global platform did not, at the time, provide the functionality that allows members to comment directly on documents; thus, it required extra work from them in that they needed to sign in to Voice Global, read the document, and then comment on it in a new thread or forum. Thus, it did not do what we intended it to do. Instead, we added the PPAG contributors to the consortium’s Microsoft SharePoint drive (Microsoft Corp). We uploaded documents to be reviewed by the PPAG contributions and researchers concurrently and in the same place, thus providing greater visibility of the insights and contributions to all. In addition, to support the PPAG contributors who did not wish to comment directly on documents in Microsoft SharePoint because of a lack of confidence in the technology, the PPAG contributors were provided with the option to (1) comment directly on Microsoft SharePoint; (2) respond to the PPIE lead directly via comments in email; or (3) comment directly in a Word document, which was then returned to the PPIE lead. If they chose option 2 or 3, the PPIE lead inserted the PPAG contributors’ comments to Microsoft SharePoint on their behalf, making it clear who the comment was from. Decision-making was, therefore, done collectively, depending on the task at hand. The researchers leading each task would review all comments from other researchers and the PPAG contributors alike, and changes to protocols or plans were discussed in meetings and shared with all the stakeholders within that group in email format. The PPIE lead also directly followed up with the PPAG contributors to highlight where their suggestions have been integrated.

### PPIE Action Plan

A refined action plan of PPIE activities was collaboratively developed by the PPIE Board and PPAG, which outlined tasks until the completion of the consortium in 2024. A comprehensive and cross-cutting approach was adopted, where the members of each work package were asked to identify completed or planned tasks that complemented the overall aims of the project. The activities were to complement the objectives by focusing on understanding patients’ perspectives on the use of digital technology and mobility assessments and to ascertain patients’ acceptance of digital mobility outcomes. Furthermore, we sought to collect patient insights into the use of digital health data, including their presentation and visualization, and to progress the learnings derived from Mobilise-D by disseminating the results across a range of stakeholders. These were then merged into an overall plan by the PPIE Board, which mapped the tasks to the consortium objectives and expectations; aligned them with National Institute for Health and Care Research PPIE guidelines; and outlined whether they were linked to management processes, research tasks, or dissemination activities (Figure 4). This plan was then refined and sent to the PPAG and the entire Mobilise-D consortium for comments and sign off.

Combined, the established PPIE structures and the action plan supported the creation of working groups. These working groups focus on groups of activities that were clustered together into key topics of interest. The researchers in the consortium and PPAG contributors were invited to identify the topics they wished to get involved in, and subgroups were developed to ensure that targeted activity toward these key areas could take place (Figure 4). Since 2022, a year after the PPIE Board and PPAG were established, these topics act as the agenda points for future PPIE Board and PPAG meetings, wherein updates are provided on each subgroup.

### PPAG’s and PPIE Board’s Contributions to and Impact on Mobilise-D

As a result of the collective work of the PPIE Board and the PPAG, a number of contributions and impacts have already been made or are being made (Table 1). Their work and input spanned management activities in relation to the running of the project, dissemination tasks for sharing the results and progress, and research-based activities for enhancing the TVS or CVS along with additional discrete tasks relating to substudies. Specifically, the PPAG contributors supported the design of additional, discrete activities, including a study on patient preferences regarding the visualization of mobility data over time, the design of a public webinar on the importance of walking, and its copresentation to people [38]. Their input was used to alter the content and wording of the public-facing summary of the TVS results and for the development of an exit survey for the participants in the CVS. In addition, their advice is sought in relation to the sustainability and next steps of the project, including future funding opportunities.

**Figure 5:**
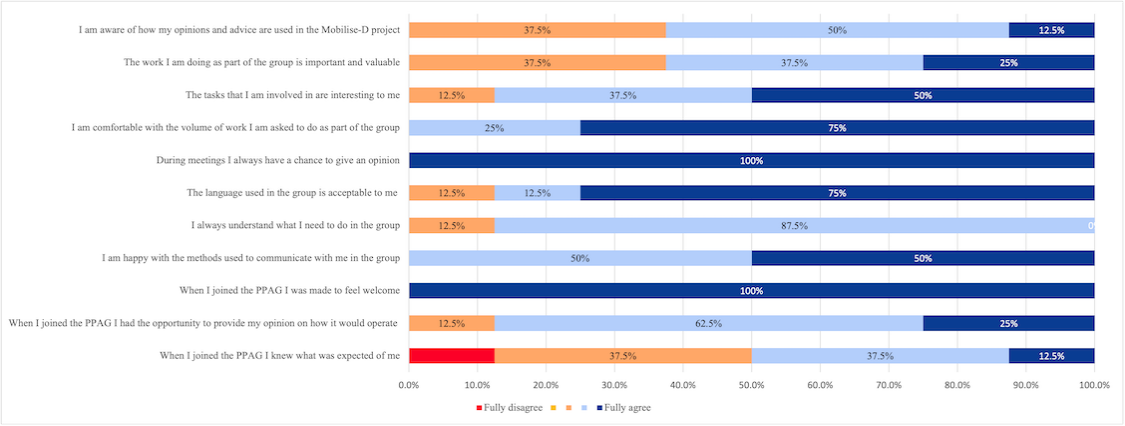
Perceptions of Patient and Public Advisory Group (PPAG) contributors as to how the group currently works.

In addition to assessing the impact of PPIE on the consortium, we also assessed the impact of belonging to the PPAG among the members after 12 months. An anonymous questionnaire was sent to the contributors to gather their feedback on a series of 5-point Likert scale questions. In addition, 3 open-ended questions asked them to detail what they liked, what they disliked, and what they wished to change about the PPAG. The findings are summarized in in Figure 4 and Table 2. According to the results, the PPAG contributors appear to be broadly satisfied with how the group works, how communication takes place within it, and the volume of work that they are asked to complete (Figure 5). This was further supported by their comments (Table 2). The areas where most development is needed appear to be around clarifying the impact of the work that the contributors complete and clearly explaining the purpose of the work that is being undertaken by both the group and Mobilise-D in general. However, 40% (3/7) of the respondents did not report any aspect of the group that they wished to change.

**Table 1:** List of the outputs and impacts of the patient and public involvement and engagement (PPIE) work of Mobilise-D to date.

Output name and details of this work				Impact
**Management activities**				Structural changes to PPIE activities within Mobilise-D and increased visibility of PPIE work
	Reimbursement document	The contributors reviewed and signed off on the reimbursement procedures in place for PPIE activities within Mobilise-D. These included the rate of pay, methods of reimbursement, and clarity of the document before its official implementation.		
	Committee membership	Members of the PPAG^a^ sit on key governance committees within Mobilise-D, including the scientific committee and executive committee (Figure 3).		
	PPAG web page	Information regarding the PPAG members and the work of the group was placed on the web. Members identified questions to include in their profiles that were of interest to them [39].		
	PPIE Action plan	The PPAG members were asked to comment on whether planned tasks were meaningful to them, that is, on whether these targeted topics of importance and provided opportunities for their active involvement.		
**Research activities**				Production of coauthored manuscripts, changes to the study outcomes, and co-design of upcoming research activities
	Conceptual framework of walking	The PPAG contributors supported the interpretation of results on a key research output around the perception and experience of real-world mobility from the patient perspective. The contributors coauthored the manuscript [29].		
	Minimally important difference questions	The PPAG provided feedback and suggested changes for the questions used in the clinical validation study, and these were implemented in the study outcomes.		
	Technical validation study results	The PPAG members supported us in developing a public-facing document that summarizes the technical validation study results using plain language. PPAG feedback included advice regarding the language used, the format of the document, and the visual tools used.		
	Exit survey	The PPAG members provided suggestions on what questions to include in the exit survey for the clinical validation study. They identified areas of priority and ways in which to structure the questions.		
	Data visualization	Work is ongoing regarding how best to visualize and communicate data to patients and members of the public. The PPAG contributors are reviewing items for approval before dissemination and advising on how data should be presented. They supported the design of a study for exploring patient preferences for the visualizations of mobility data over time.		
	Data sharing	Work is ongoing to design tasks around the topic of data sharing. Contributors are providing insight into the topics of importance in this area.		
	Digital inclusion	Work is ongoing to design tasks around the topic of digital inclusion.		
	Future funding applications	The PPAG contributors have been involved in and are named collaborators in additional grant applications that seek to develop certain aspects of Mobilise-D further.		
**Dissemination activities**				Increasing the engagement with and awareness of Mobilise-D and PPIE activities
	PPIE learning points	In preparation for this paper, the PPAG members were asked for their input on the design and layout of the paper, and they provided feedback regarding their experiences within the PPAG and coauthored this manuscript.		
	Public handbook	Codeveloped a comprehensive web-based handbook that outlines the aim and purpose of the consortium and briefly describes the technical and clinical validation studies in a manner that can be easily understood by the public. This acts as an important guiding document for any new PPAG members.		
	TOPRA^b^ discussion	The PPIE structures were presented to TOPRA in France in November 2021 by the members of the PPIE Board and PPAG to outline how patients and researchers can work together.		
	Conference abstract	A panel discussion was arranged for The Professional Society for Health Economics and Outcomes Research in December 2021 with the researchers and PPAG members; unfortunately, COVID-19 concerns and restrictions meant that the panel members were not able to attend this event.		
	Public webinars	The PPAG designed a webinar that aimed to highlight to clinicians and researchers why mobility is important. The PPAG members sat on the discussion panel with Mobilise-D clinicians [38]. We also have patient representatives sitting on the discussion panel of cohort-specific webinars detailing how the study is being run and what we know about digital mobility outcomes with respect to each cohort.		
	Invited discussion	The PPAG members and researchers in Mobilise-D spoke at “Reverse Engineering of Digital Measures—A Conference on Patient-Centric Digital Evidence Co-organized by the ETH Zurich and FNIH” in September 2022 to outline Mobilise-D and how researchers and patients collaborate in the development of digital outcomes.		
	Conference support	A PPAG contributor supported the researchers at the 2023 World Parkinson Congress, where Mobilise-D had a research stand. They worked alongside the researchers to inform the attendees of the consortium and some upcoming activities.		
	Public videos of the results	The PPAG contributors are currently working alongside the researchers to support the design of a public-facing video and infographic outlining some seminal patient-centered results from Mobilise-D		

^a^PPAG: Patient and Public Advisory Group.

^b^TOPRA: The Organisation for Professionals in Regulatory Affairs.

**Table 2:** Patient and Public Advisory Group (PPAG) contributor open-ended question feedback.

Domain			Contributor feedback
**Areas of the PPAG they like**			
	Providing a valuable contribution	“Being able to participate in the project by contributing my own experiences easily. The tools and mechanisms to do so are very effective and the PPAG is managed excellently.”	
	Providing a valuable contribution	“I feel like I may be contributing (in some small way) to improving the understanding (by doctors etc) of patient’s needs in these areas so helping future diagnosis and assistance.”	
	Providing a valuable contribution	“I think that it is very important to have a chance to communicate about this theme, to provide our experience as patient but also being representative of our patient community. I like the diversity in our group and the different experiences collected to create this work. I like that the importance of the patient’s perspective is central for this work.” “Being able to contribute to an outstanding research project. The variety of tasks makes the participation truly interesting. The group is managed very well, tasks are explained and there is always enough time to deliver.”	
	Sense of belonging	“I feel that communication is excellent. As a layman person, I feel that things are being explained in a simple manner, and if I do not understand something I am comfortable asking for an explanation. Mobilise-D professional group is very accommodating and enabling.”	
**Areas of the PPAG where change is needed**			
	Lack of clarity around impact	“I’m not always sure at the end of a meeting if I have contributed or if I’m expected to do something”	
	Lack of clarity around impact	“I would like a clear end of meeting summary (by the Chair) as to what is happening next and who is expected to do what.”	
	Diversity of people	“Being English the language used, it left a lot of people out of the process. It is not easy and I don’t know if it is affordable to have a summary of the most important documents in 2-3 more languages. It will provide also other perspectives.”	
	Uncertainty on study aims	“Sometimes, not being a clinician or researcher, a feeling of not quite understanding exactly what is trying to be accomplished.”	

### Lessons Learned and Key Recommendations

#### Overview

This section outlines the key learning points and recommendations developed based on the processes described earlier (Textbox 1).

**Key recommendations for the establishment of patient and public involvement and engagement (PPIE) structures in complex, multicohort consortia.**

1. Researchers should be educated on the impact of PPIE and recognize that it is fundamental to all stages of the research cycle.
  Some research members of the consortium may need to undertake PPIE training to highlight the importance of the work and support ways in which it can be undertaken at all stages of the research process. It is important to complete this early to ensure that PPIE is meaningful from the beginning of any project. We suggest that researchers in consortia receive training in this area before commencing the project. Furthermore, many universities are beginning to develop more formal PPIE networks or advisory centers. These should be engaged with to highlight important areas to consider when developing PPIE frameworks, plans, and structures.
2. PPIE work needs to be an integrated, consortium-wide effort and considered as early as possible to be impactful. Ideally, this will occur in the project design phase.
  Consortia should embed PPIE pathways within their structures during their development. Indeed, PPIE should be included in the design of the project and during the initial funding application. This lays the foundations from which PPIE tasks will take place throughout the project and will support their continued evolution and development as the consortium progresses.
3. Consortia should employ an individual whose main role is to manage and co-ordinate PPIE activities.
  Consortia should have a dedicated PPIE leader who facilitates and establishes links between Patient and Public Advisory Group (PPAG) members and the rest of the consortium. However, even with this person in place, activities must occur with the support and ownership of all work packages and consortium partners, rather than simply a small number of PPIE champions. PPIE leaders must have the capacity and resources to carry out the work; must be skilled in facilitating groups, building trust, and managing challenging conversations (particularly as medical conditions can be associated with trauma); and must be able to communicate effectively. Ideally, this person should have undertaken training in how to conduct PPIE and be from a background that is comfortable engaging with patients and public, for example, through qualitative research or clinical work. The relationship that PPIE leaders build with advisers is integral to their engagement with the research. Furthermore, previous experience in managing PPIE should be prioritized. That being said, research groups must recognize that all researchers can be trained in this area; thus, in the event that such a person cannot be identified, researchers should work to develop these skills in people within their projects and reach out to more experienced or established PPIE leads for mentoring and advice.
4. All partners need to be prepared to refine and iterate the PPIE strategy, structures, or plans while the project is evolving.
  For PPIE work to be meaningful, it is important that it is both inclusive and flexible. This means that consortia partners must be prepared to react to changes that may occur in their plans and timelines, along with their PPIE structures. For example, patient advisers may highlight questions that they feel are important to answer that researchers had not planned for, which may require additional tasks to be planned or methods for conducting PPIE tasks to be altered. Furthermore, patient advisers and consortium partners may face changes to their personal circumstances that require them to be replaced within the PPIE structures.
5. Plan for the use of resources early to allow for the full scope of work
  Consortia must be prepared to plan for PPIE as well as budget the use of resources for PPIE. For example, the use of additional platforms to support communication and engagement, the inclusion of full-time PPIE facilitators, and PPAG member expenses should all be included in project budgets. Additional resources will be required to develop and conduct tasks such as surveys, webinars, and focus groups. If consortia fail to budget for these tasks accordingly, it may be difficult for them to be operationalized or may impact their potential to be impactful.
6. Establish clear terms of reference with regard to the scope of both the project overall and the PPIE work specifically
  It is important that PPAG advisers understand the scope of the project so that they are aware of its limitations and what is and what is not possible within its lifetime. Most research projects will hope or aim for their tool, intervention, or research outputs to be used in future trials or that they may change health care in the future. There is sometimes a big gap between this potential and what will be developed. This needs to be clear for contributors from the start so as to avoid the potential for frustration. Within this context, it needs to be clear as to what type of activities fall under the scope of the PPIE structures. The purpose of this is not to exclude ideas and iterations beyond what were planned but to be aware of what activities fit within the overall scope of the project.
7. Plan for research tasks in line with the objectives but considering the additional steps required within the PPIE structures, which is recommended to be done through a strategic framework
  Successful and meaningful PPIE activities require a strategic framework that outlines the principles and structures of the PPIE activities within the context of the specific project. Consortia need to be clear about the aims of PPIE, how it will operate within their consortia, what topics it will cover, and how it will be implemented. Here, it needs to be recognized that meaningful PPIE requires significant time to ensure that they are planned for and conducted in a manner that supports open collaboration. This can be a difficult balance, as patients may identify clear priorities that are transformative to the consortium while still remaining within the scope of the funded work. Consequently, setting out clear structures that outline how changes to scope will be made is important. The time commitment required for this planning is substantial and needs to be included within research project timelines. Outlining projects and their objectives in full during the initial development of a PPAG may support collaboration. For example, patient contributors may need to be aware that funding is provided to complete a specific objective and that, therefore, consortia may sometimes have the scope to address only some, and not, of the identified needs. Similarly, project management must be open to the potentially transformative effects of PPIE work on planned activities and must be prepared to alter plans when the alterations are within scope, link to research objectives, and are important changes advised by PPAG contributors. In addition, time is needed to ensure that patient contributors have sufficient opportunity to review documents before meetings. Researchers must, therefore, adjust their timelines so as to not delay work or place undue pressure on contributors. For example, a 2-hour PPAG meeting requires double that time when administration and the development of preparation documents are considered. Following the meeting, further time is required to summarize and document the minutes of the meeting and feed these minutes back to the PPAG itself and the wider consortium. Failure to consider this time may result in delays, frustrations on the part of both researchers and PPAG members, a lack of clarity regarding expectations, feelings of overwork without impact, and potentially less impactful meetings [9].
8. Be mindful and aware of potential digital exclusion
  There will be times when full inclusivity may not be possible; however, researchers must be aware of this, work against it where possible, and understand the implications of this for their work. Although there is rarely a right or wrong answer to what method of meeting and practice works best, researchers need to weigh up the benefits, risks, and needs of the patient contributors they are engaging with. They should determine the best methods for communicating with contributors and ensure that contributors have the skills and supports to be able to engage with the research process. When limitations are unavoidable, methods should be built to counter these limitations in PPIE plans.
9. Establish clear terms of reference and agreed code of conducts with contributors in the first meeting and review them for effectiveness frequently
  Detailed documents outlining the expected roles, responsibilities, and conduct are important to establish trust and boundaries. These also support both researchers and contributors in autonomously deciding how they want to work and what practical arrangements work best for them. Because of these documents, agreed processes were established and implemented within Mobilise-D. To further support open discussion in and between meetings, documents were sent 1 month in advance of the meeting to allow members adequate time to read material and consider any questions and comments that they may have on the material. It was also imperative that the documents be provided in a succinct manner but that they provide sufficient information to support full interpretation. Furthermore, members are given multiple ways in which they can provide feedback so that they can choose which suits them best. Finally, it was noted in the first meeting that PPAG members are invited to get involved in any number of activities that they wish to take part in or have an interest in. The terms of reference and code of conduct provided the foundations from which such processes could be built upon.
10. Allow for flexibility with PPAG membership and invested resources
  To ensure continuity in discussion, it is important to have a small number of people in each group who are often present. However, a certain amount of flexibility and support is required for those who may enter the process later or return after a break. Individual meetings with the PPIE lead (AK) are arranged for anyone who joined the PPAG after the initial meeting, where expectations are discussed and an introduction to the group, its work, and the work of the wider consortium is provided. Furthermore, those who miss meetings are provided with the link to the recording should they wish to see it, and individual calls are arranged with members at any time should they wish to discuss a topic in further detail.
11. Develop a consortium handbook for the public
  Mobilise-D is a comprehensive project with multiple stakeholders, aims, and components. PPAG members highlighted that the small number of slides (n=4) that was provided before joining the project did not provide enough detail to support the understanding of the project. Thus, it is recommended that research consortia develop a handbook for members of the public, which clearly outlines the project’s aims, structure, planned studies, and desired outcomes and where contributors may engage. This document should be comprehensive but easily understandable.
12. Evaluate and document the impact of PPIE work from multiple stakeholders’ perspectives and refine the work based on any arising needs
  There is no single best way to measure the impact of PPIE work. However, some areas of consideration include determining the impact on research agendas, research design, those involved, the wider community, and future plans [7]. Thus, researchers should document what recommendations were made by patient representatives, what changes were made in response to these recommendations, and what outcomes were observed [7]. Within Mobilise-D, we have begun to document the tasks and areas to which the PPAG has contributed, beyond just creating publications that fail to account for the full breadth of the work of the group (Table 1). In addition, we have reviewed the PPAG members’ perspectives on how the group is working. Evaluations such as this need to continue for the remainder of the consortium to derive a complete picture of how these structures and this work have impacted the work of Mobilise-D.

#### Recognizing the Need for a Consortium-Wide Strategic Approach

Although ad hoc interactions can meet short-term PPIE needs, partnerships require frequent, planned, and consistent interactions [20]. A key learning that was formed early within the consortium was the recognition that meaningful PPIE requires a clear strategic framework and actionable implementation to be in place. For us, meaningful PPIE is PPIE where all contributors (including patients and carers) are listened to and all their voices are included in the decision-making processes with a focus on topics that are important to patients. It is operationalized through the development of PPIE structures, as outlined subsequently, and through the recognition of the impact of PPIE by tracking the changes and ideas implemented because of PPIE. This first and foremost requires those involved in the governance of a project to be aware of and endorse not only the importance of this work but also the fact that they know how to conduct it. Although it may appear to be relatively easy to include patient representatives in a project, truly embedding their contributions in a way that shapes research plans and outputs requires consortium partners to understand and acknowledge the importance of this work [40]. As a result, the PPIE structures of Mobilise-D underwent significant changes between 2019 and now. This was to avoid the pitfalls of potentially superficial PPIE tasks or inadvertently missing tasks that were important to both the project and patients. Consequently, the key recommendations from this step are as follows (Textbox 1):

1. Researchers should be educated on the impact of PPIE and recognize that it is fundamental to all stages of the research cycle.
2. PPIE work needs to be an integrated, consortium-wide effort and considered as early as possible to be impactful. Ideally, this will occur in the project design phase.
3. Consortia should employ an individual whose main role is to manage and coordinate PPIE activities.
4. All partners need to be prepared to refine and iterate the PPIE strategy, structures, or plans while the project is evolving.

#### Creating PPIE Structures

There are acknowledged barriers to conducting meaningful PPIE, including limited funding and expertise, time constraints, and the challenges in recruiting patient representatives [19]. These extend to the creation of PPIE structures. The structures listed earlier took up to a year to fully embed into the consortium and begin functioning effectively. Critical to this was ensuring that the contributors were aware of the scope of the project and had their expectations of this met. In truth, achieving this was difficult, as some contributors highlighted that they were unsure of how far the work of Mobilise-D would extend, causing them some frustrations. Furthermore, patient engagement through PPIE structures can have a transformative impact on research plans. This can be challenging, as consortia need to balance the identified preferences and needs of patients with the planned scope of funding. Thus, the establishment of terms of reference, priority setting, group goals, and a description of roles and responsibility can support enhanced understanding when initially creating PPIE partnerships and groups [21]. The time spent developing the PPIE structures was significant but nonetheless worthwhile. Thus, a robust and inclusive project development process should be undertaken to mitigate some of these challenges, key recommendations for which are as follows (Textbox 1):

5. Plan for the use of resources early to allow for the full scope of work

6. Establish clear terms of reference with regard to the scope of both the project overall and the PPIE work specifically

7. Plan for research tasks in line with the objectives but considering the additional steps required within the PPIE structures; which is recommended to be done through a strategic framework

#### Developing the PPAG and Its Operations

Making research inclusive of a range of people from various sociodemographic, ethnic, educational, cultural, health, and age range backgrounds is a key aim of PPIE work [22,40]. Within a large international consortium such as Mobilise-D, some of the notable limitations of our structures include the need for the PPAG members to speak English and for meetings to be conducted on web-based platforms. The reliance on English was highlighted as a limitation by our PPAG members; however, this is also a limitation present in regulatory agencies working with PPIE contributors [41]. In response, the consortium sought to enhance inclusion in other PPIE activities by engaging with the Voice Global platform to advertise opportunities in multiple languages. The Mobilise-D website has translation functions built into it, and the consortium collaborates with patient societies across Europe to support greater reach. Furthermore, previous reports have noted that liaising with trusted advocates or gatekeepers is an important way to engage with contributors from underserved groups [42]. Although we acknowledge the limitations in the backgrounds of our PPAG contributors, we nonetheless suggest that other groups consider such strategies to not only recruit diverse contributors but also support cultural differences in a more globally represented group.

Nonetheless, inclusivity in the digital world goes beyond simply ensuring that contributors come from varied backgrounds. Within Mobilise-D, the global spread of contributors resulted in a decision to rely on web-based methods of communication, including video calls. The COVID-19 pandemic may have inadvertently supported PPIE work across consortia. As travel became more difficult for everyone, especially for those who were susceptible or immunocompromised [43], many people were forced to embrace this form of technology quickly. Thus, remote meetings may support inclusivity by providing a safe environment for people to engage in research [43]. However, web-based interaction risks digital exclusion through less natural conversation, unpredictable internet connections, the removal of nonverbal cues, and the increased pressure on facilitators to manage discussions [43-45]. Within Mobilise-D, the creation and coagreement of the code of conduct and terms of reference were essential for creating a safe environment by ensuring that the researchers planned activities and meetings appropriately, including the substantial time required for this. These documents helped align expectations and provide a framework for how all the PPAG contributors, regardless of their culture or native language, could be supported to contribute. In the first meeting of the group, the contributors agreed to these terms, thus supporting their buy-in. Furthermore, the contributors knew what to expect in meetings, had a clear contact point to communicate with, and were provided with information in advance of meetings according to their preferred timelines. At times, some of the agreed communication methods needed to be emphasized within meetings to ensure that everyone’s voice was heard, whereas PPAG feedback demonstrated that the researchers still needed to improve their communication around certain aspects of the group. Furthermore, despite all this preparatory work, the PPAG highlighted that not enough easily understandable information regarding the work of Mobilise-D had been provided in advance; thus, there was a need for us to produce a consortium handbook to aid the understanding of the complex project for the contributors who were joining the project for the first time.

Finally, as with any group, membership changes occurred throughout the first year. On the PPIE Board, members may depart when their contract ends or if their personal circumstances change, whereas on the PPAG, changing personal circumstances resulted in some members needing to take a break from the group or step away altogether. The challenge is to ensure that the procedures in place support individuals to remain involved if they wish while not overburdening them. Thus, considering these experiences, the following recommendations can be made regarding operationalizing PPIE structures and activities:

8. Be mindful and aware of potential digital exclusion

9. Establish clear terms of reference and agreed code of conducts with contributors in the first meeting and review them for effectiveness frequently

10. Allow for flexibility with PPAG membership and invested resources

11. Develop a consortium handbook for the public

#### Evaluating Impact

One of the criticisms of PPIE is that its impact is often perceived as unclear, a factor highlighted by our PPAG contributors as well [9]. Researchers are used to establishing impact through quantitative and experimental data analysis, publication outputs, and trial recruitment and retention rates [1,9]. However, impact is highly dependent on the context of each project, along with the baseline skills of researchers conducting the study and the tasks that patient representatives were involved in [7]. Consequently, consortia need to be aware that impact goes beyond traditional quantitative outcomes and should include the experiences and perspectives of all stakeholders within the project, as suggested within the key recommendation of this step:

12. Evaluate and document the impact of PPIE work from multiple stakeholders’ perspectives and refine work based on any arising needs

#### Conclusions

This paper highlighted the work undertaken to set up PPIE structures within a large European consortium to promote and support the occurrence of meaningful and efficient patient and public involvement related to the creation of digital mobility outcomes. The structures and work outlined in this paper provided the Mobilise-D consortium with a foundation from which future PPIE tasks can be created and managed with clearly defined collaboration between researchers and patient representatives across Europe. This may be used as a template for future consortia to follow and learn from.

## Abbreviations

CVS: clinical validation study
PPAG: Patient and Public Advisory Group
PPIE: patient and public involvement and engagement
TVS: technical validation study
